# Association of mitochondrial DNA haplogroup and hearing impairment with aging in Japanese general population of the Iwaki Health Promotion Project

**DOI:** 10.1038/s10038-022-01011-6

**Published:** 2022-01-17

**Authors:** Shiori Miura, Akira Sasaki, Shuya Kasai, Takayuki Sugawara, Yasunori Maeda, Shinichi Goto, Takashi Kasai, Nami Shimizume, Songee Jung, Takuro Iwane, Ken Itoh, Atsushi Matsubara

**Affiliations:** 1 Department of Otorhinolaryngology, Hirosaki University Graduate School of Medicine, Hirosaki, Japan; 2 Department of Stress Response Science, Hirosaki University Graduate School of Medicine, Hirosaki, Japan; 3 Research Institute of Bio-System Informatics, Tohoku Chemical Co., Ltd, Morioka, Japan; 4 Center of Innovation Research Initiatives Organization, Hirosaki University, Hirosaki, Japan; 5 Department of Digital Nutrition, Graduate School of Medicine, Hirosaki University, Hirosaki, Japan; 6 Hirosaki University COI Research Initiative Organization, Hirosaki University, Hirosaki, Japan

**Keywords:** Epidemiology, Risk factors

## Abstract

Age-related hearing loss (ARHL) is a complex multifactorial disorder. Studies in animals, including mitochondria-mutator mice, and in human suggest that oxidative stress and mitochondrial disturbance play an important role in the pathoetiology of ARHL. Mitochondrial DNA (mtDNA) haplogroups are populations with genetically similar traits, and they have been reported to affect the mitochondrial function of oxidative phosphorylation. To gain further insights into the relationships between mitochondrial haplotypes and the susceptibility to cochlear aging, in this study, we aimed to elucidate how the differences in mtDNA haplogroups may affect ARHL development in Japanese general population. We focused on early onset ARHL, as the same mtDNA haplogroup can show either a negative or positive effect on systemic co-morbidities of ARHL that appear later in life. A total of 1167 participants of the Iwaki Health Promotion Project were surveyed in 2014, and 12 major haplotype groups (D4a, D4b, D5, G1, G2, M7a, M7b, A, B4, B5, N9, and F) were selected for the analysis. A total of 698 subjects aged 30 to 65 years were included in the statistical analysis, and the hearing loss group consisted of 112 males (40.3%) and 111 females (26.4%). Multiple logistic regression analysis showed that the male subjects belonging to haplogroup A had a significantly increased risk of hearing loss, whereas the female subjects belonging to haplogroup N9 had a significantly decreased risk of hearing loss. These results suggested that the mtDNA haplogroup may be an indicator for future risk of morbidity associated with ARHL.

## Introduction

Hearing loss is the most common sensory disorder in the world. Aging is the main cause of hearing loss, affecting tens of millions of people worldwide [1]. In 2008–2010, the prevalence of age-related hearing loss (ARHL) was 71.4% in males and 67.3% in females aged 75 years and above in Japanese population [2]. The loss of hearing sensitivity begins at the highest frequencies, and it has an adverse effect on understanding speech in the elderly population [3]. Hearing loss may be causally related to dementia, possibly through cognitive reserve exhaustion, social isolation, environmental deafferentation, or a combination of these processes [4].

The World Health Organization defined hearing loss as a pure tone average of more than 25 dB thresholds at 500, 1000, 2000, and 4000 Hz [1], and the prevalence rate is reportedly 73% in individuals over 70 years of age in the Beaver Dam cohort [5]. Therefore, it was estimated that most individuals older than 70 years have hearing loss. Furthermore, it has been shown that hearing thresholds at 10,000 Hz increased in subjects older than 30 years, especially in males [6], and hearing loss at high frequencies with aging is common [7, 8]. Thus, it is suggested that people over 30 years of age experience hearing loss at high frequencies.

Besides aging, ARHL is also associated with a variety of factors, including noise exposure [9], ototoxic drugs [10], malnutrition [11], smoking [12], hypertension [13], and type 2 diabetes [14]. Yamasoba et al. proposed a conceptual model of ARHL, in which these environmental and individual genetic factors cause metabolic stress in inner ear cells and produce reactive oxygen species (ROS) in the mitochondria [1]. ROS cause oxidative damage, which accumulates over time and leads to tissue dysfunction during aging [1]. ROS attack mitochondrial membranes and mitochondrial DNA (mtDNA) near the site of their formation, leading to mitochondrial dysfunction [15], which may lead to tissue dysfunction in all somatic systems.

Human mtDNA and mtDNA, in general, are maternally inherited and not affected by homologous recombination during gamete formation, unlike nuclear chromosomal DNA [16, 17]. Therefore, mutations in mtDNA in the reproductive tissues of maternal lineages are directly inherited by the offspring. mtDNA mutations have accumulated and diverged in human mtDNA phylogenetic trees. Phylogeographic studies on human mtDNA have shown that we can trace the maternal origin of mtDNA to the first human ancestor “mitochondrial Eve” in Africa along with its movement trajectories in the historical time period. An mtDNA haplogroup is a population of the same mitochondrial lineage that shares a set of mtDNA variants. In fact, studies have reported a correlation between specific mtDNA haplogroups and type 2 diabetes [18], myocardial infarction [19], and atherothrombotic cerebral infarction [20].

In this study, we aimed to elucidate how the differences in mtDNA haplogroups affect the development of ARHL in Japanese population.

## Materials and methods

### Subjects

**Fig. 1:**
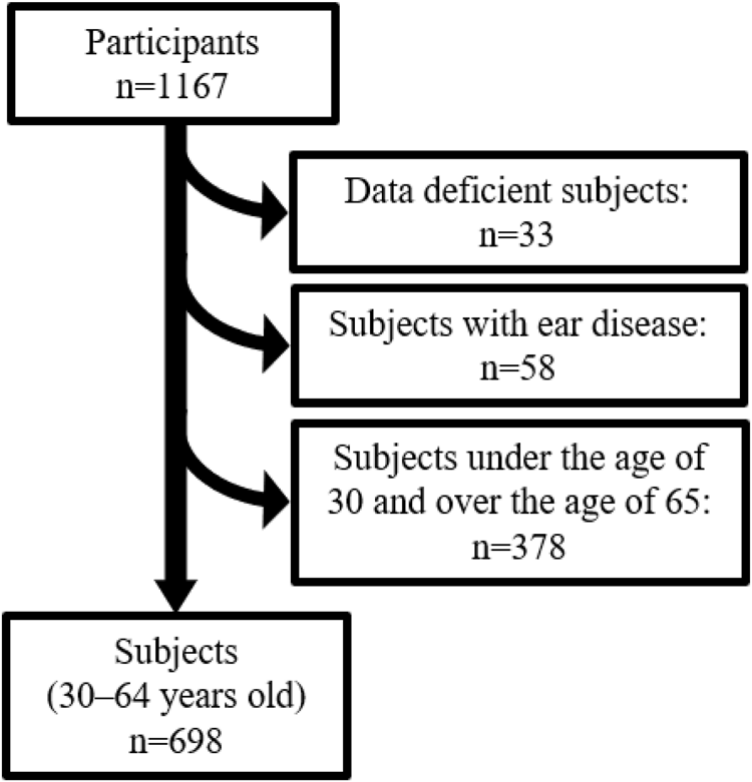
Flow chart illustrating the selection of subjects There were 698 individuals aged 30 to 64 years; 33 data-deficient individuals, 58 individuals with a history of ear disease, and 378 young and elderly individuals were excluded from our analyses

The Iwaki Health Promotion Project is an annual, large-scale epidemiological survey performed in Iwaki District, Hirosaki City, Japan. All residents older than 20 years of age living in this district are invited to participate in the project. The data collected during this project in 2014 were used in the study. A total of 1167 participants were surveyed, among which 33 data-deficient subjects and 58 subjects with ear disorders, including acute sensorineural hearing loss, Meniere’s disease, cholesteatoma, and traumatic inner ear disorder, were excluded. To detect early hearing loss with aging, subjects aged only 30‒64 years were enrolled in the present study. Finally, 698 subjects were included in the statistical analysis (Fig. 1).

Data collection and genetic analysis in the present study and the Iwaki Health Promotion Project were approved by the Ethics Committee of Hirosaki University School of Medicine (authorization numbers: 2014-014, 2014-377, 2016-028), and all subjects provided written informed consent before participating in the project.

### Lifestyles and laboratory data

To obtain the participants’ lifestyle information, we used a self-administered questionnaire and conducted face-to-face interviews to determine the medical history, drug information, noise exposure history, smoking habit, and drinking habit of the participants. The pack-years (number of cigarette packs per day × years of smoking) for smoking habit was also calculated. Venous blood samples were obtained early morning with the participants on an empty stomach to examine hemoglobin A1c (HbA1c), triglyceride (TG), high-density lipoprotein cholesterol (HDLC), and low-density lipoprotein cholesterol (LDLC). The diseases that may affect hearing were defined as follows: hypertension was defined according to self-reported physician diagnosis and current use of antihypertensive medicine; diabetes mellitus was defined according to self-reported physician diagnosis and current use of antihyperglycemic medicine or the HbA1c level greater than 6.5% according to the diagnostic criteria of the Japan Diabetes Society; dyslipidemia was defined according to self-reported physician diagnosis and current use of lipid-lowering medicine or serum level of TG ≥ 150 mg/dL and/or HDLC < 40 mg/dL and/or LDLC ≥ 140 mg/dL according to the diagnostic criteria of the Japan Atherosclerosis Society.

### Audiometric assessment

Pure tone audiometry was conducted by trained doctors and technologists in a quiet room. Using audiometers (AA-73A; RION Co., Japan), they measured air-conducted hearing on both sides at 125, 250, 500, 1000, 2000, 4000, and 8000 Hz. The worse-hearing ear was used for the analysis, depending on the average thresholds at 500, 1000, 2000, and 4000 Hz. As the beginning of hearing loss with aging occurs at high frequencies [7, 8], average hearing thresholds at 4000 and 8000 Hz were used for analysis. The subjects with average hearing at 4000 and 8000 Hz greater than 25 dB were included in the hearing loss group, and the remaining subjects were included in the control group.

### Genetic analysis

Genomic DNA extracted from venous blood samples was subjected to whole-genome sequencing by Takara Bio Inc. (Shiga, Japan). Sequence mapping was performed at the Institute of Medical Science, University of Tokyo. The mitochondrial haplogroup of each BAM file of the mitochondrial genome was assigned using MitoSuite version 1.0.9 [21].

### Statistical analysis

The characteristics of the subjects (males and females) and the hearing loss and control groups for each gender were compared using Student’s *t*-test and chi-square test. A comparison of specific mtDNA haplogroup prevalence was performed using the chi-square test.

Risk factors influencing high-frequency hearing loss were evaluated using the multiple logistic regression analysis separately for males and females. The dependent variable was the presence or absence of high-frequency hearing loss, and the independent variables were age, smoking history (pack-years), drinking habit, noise exposure, hypertension, diabetes, and dyslipidemia. Furthermore, each of the 12 haplogroups were added to the independent variable.

Test results with *p* < 0.05 were regarded as statistically significant. All statistical analyses were performed using IBM SPSS Statistics (version 25.0; IBM Corp., Armonk, NY, USA).

## Results

### Characteristics of the subjects

The characteristics of our subjects by gender are shown in Table 1a. The 698 subjects comprised 278 males (39.8%) and 420 females (60.2%). The number of subjects in the hearing loss group was 112 (40.3%) for males and 111 (26.4%) for females, and the prevalence of hearing loss was significantly higher in males than in females. In addition, the average age was significantly lower in males, but the pack-years, prevalence of drinking habit, noise exposure, and dyslipidemia were significantly higher in males.

**Table 1:** **a** Characteristics of the subjects by gender. **b** Characteristics of the subjects with hearing loss and control groups for each gender

	All subjects (*n* = 698)		
	Males	Females	*p* value
	(*n* = 278)	(*n* = 420)	
Hearing loss	112 (40.3%)	111 (26.4%)	<0.001*
Age	48.1 ± 10.4	50.5 ± 10.4	0.003*
Pack-years	16.4 ± 17.5	3.0 ± 7.2	<0.001*
Drinking habits	207 (74.5%)	146 (34.8%)	<0.001*
Noise exposure	70 (25.2%)	48 (11.4%)	<0.001*
Hypertension	43 (15.5%)	71 (16.9%)	0.62
Diabetes	21 (7.6%)	20 (4.8%)	0.13
Dyslipidemia	120 (43.2%)	129 (30.7%)	0.001*

	Male subjects (*n* = 278)			Female subjects (*n* = 420)		
	HL	Controls	*p* value	HL	Controls	*p* value
	(*n* = 112)	(*n* = 166)		(*n* = 111)	(*n* = 309)	
Age	55.5 ± 7.9	43.1 ± 8.7	<0.001*	58.0 ± 6.4	47.8 ± 10.3	<0.001*
Pack-years	21.8 ± 20.1	12.7 ± 14.4	<0.001*	2.8 ± 6.8	3.1 ± 7.3	0.71
Drinking habits	84 (75.0%)	123 (74.1%)	0.87	33 (29.7%)	113 (36.6%)	0.19
Noise exposure	35 (31.3%)	35 (21.1%)	0.06	17 (15.3%)	31 (10.0%)	0.13
Hypertension	28 (25.0%)	15 (9.0%)	<0.001*	29 (26.1%)	42 (13.6%)	0.003*
Diabetes	13 (11.6%)	8 (4.8%)	0.04*	6 (5.4%)	14 (4.5%)	0.71
Dyslipidemia	48 (42.9%)	72 (43.4%)	0.93	40 (36.0%)	89 (28.8%)	0.16

Plus‒minus values are mean ± SD. Age and pack-years were examined using the *t*-test. Other variables were examined using chi-square statistics. Statistical significance was set at *p* < 0.05 in all analyses (*). *HL* Hearing loss

The characteristics of subjects in the hearing loss and control groups for each gender are shown in Table 1b. In males, the average age, pack-years, prevalence of hypertension, and diabetes were significantly higher in the hearing loss group than in the control group. In females, the average age and prevalence of hypertension were significantly higher in the hearing loss group than in the control group.

### Distribution of the mtDNA haplogroups

**Fig. 2:**
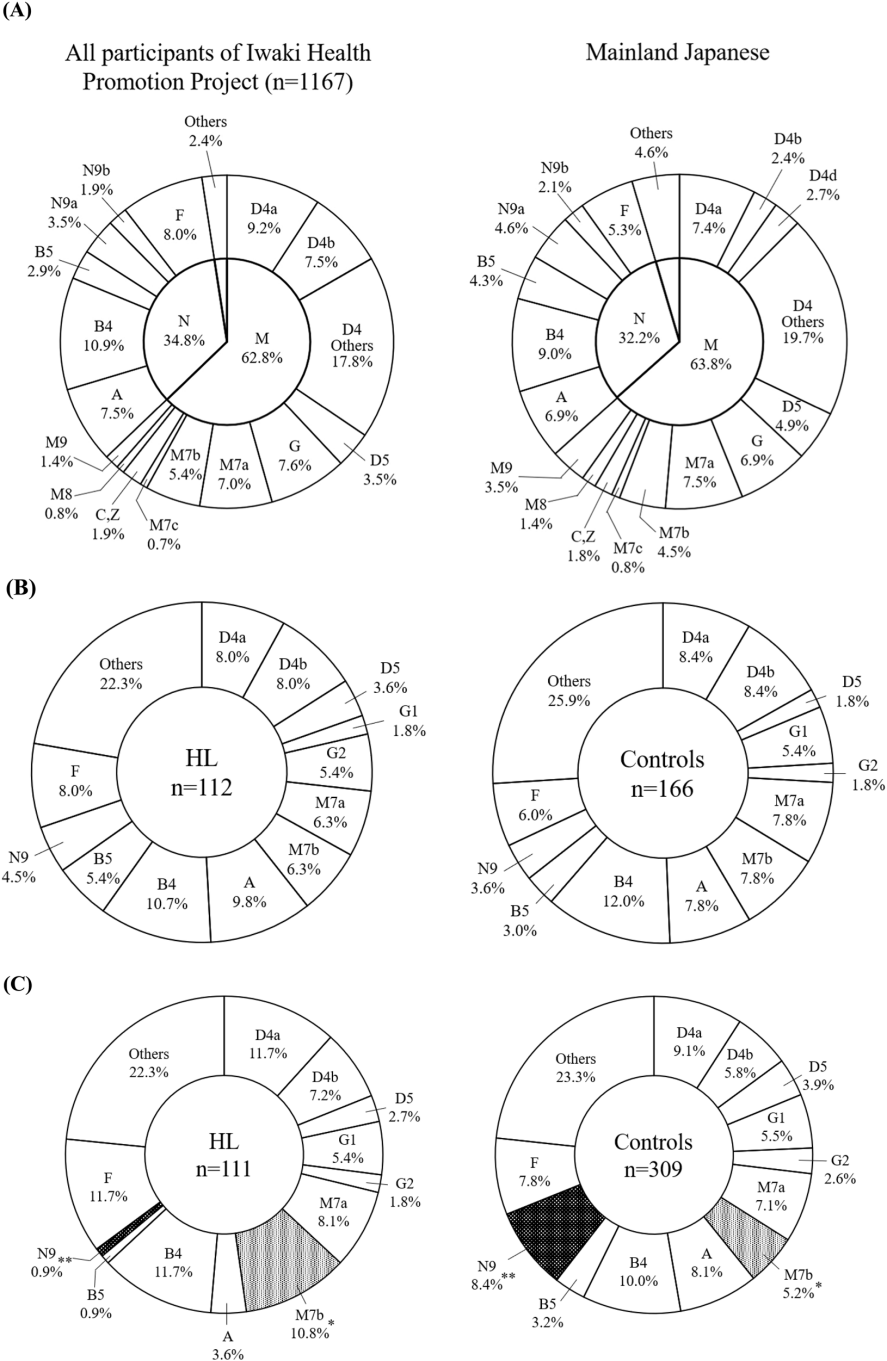
Distribution of the mtDNA haplogroups **A** Distribution of the mtDNA haplogroups in all participants of the Iwaki Health Promotion Project (*n* = 1167: left panel) was compared with that previously reported in mainland Japanese (right panel). The panel of mainland Japanese was constructed from the data from Tanaka et al. [22]. Please also refer to Tanaka et al. for the variant set for each haplotype [22]. Distribution of the mtDNA haplogroups in the subjects in question (*n* = 698) is shown as a percentage of each haplogroup in individuals with hearing loss (HL) and control groups. **B** Male subjects, **C** Female subjects. All variables were examined using chi-square statistics. Statistical significance was set at *p* < 0.05. For mtDNA haplogroups with a significant difference, the *p* value was added to the data label. *p = 0.04, **p = 0.006

The prevalence of the mtDNA haplogroup among the 1167 participants is shown in Fig. 2A (left panel). The distribution of the mtDNA haplotypes in Iwaki District was not considerably different from that in mainland Japanese (Fig. 2A, right panel) [22]. To analyze the 698 subjects in question for hearing ability, we selected 12 major haplogroups (D4a, D4b, D5, G1, G2, M7a, M7b, A, B4, B5, N9, and F) and classified haplotypes of rare frequencies as “others”. In males, there was no significant difference in the distribution of haplogroups between the hearing loss and control groups (Fig. 2B). The haplogroup M7b was significantly more common (*p* = 0.04) and haplogroup N9 was significantly less common (*p* = 0.006) in the hearing loss group of female subjects (Fig. 2C).

### Association of the mtDNA haplogroups and hearing loss

Table 2 shows the results of the multiple logistic regression analyses for haplogroup D4a independently in males and females. There was a significant correlation between hearing loss and age; however, there was no correlation between hearing loss and haplogroup D4a in both males and females. Noise exposure significantly increased the risk of hearing loss among male subjects, and dyslipidemia significantly decreased the risk of hearing loss among female subjects. There was no correlation between hearing loss and known risk factors such as smoking history, drinking habits, hypertension, and diabetes. Furthermore, the multiple logistic regression analyses after adjusting for age, pack-years, drinking habits, noise exposure, hypertension, diabetes, and dyslipidemia were performed on the remaining 11 haplogroups. The results were similar to those for haplogroup D4a, and there was no correlation between hearing loss and the other independent variables (data not shown). The results of the multiple logistic regression analyses along with the *p* value, odds ratios (OR), and 95% confidence interval (CI) for each haplogroup are summarized in Table 3. Male subjects belonging to haplogroup A had a significantly increased risk of hearing loss (*p* = 0.01), with an odds ratio of 4.096 (95% CI: 1.327–12.643) (Table 3). Female subjects belonging to haplogroup N9 had a significantly decreased risk of hearing loss (*p* = 0.02), with an odds ratio of 0.091 (95% CI: 0.012–0.712, Table 3). Haplogroup M7b was not significantly associated with hearing in this analysis.

**Table 2:** Results of the multiple logistic regression analysis for haplogroup D4a and cofounding factors that influence high-frequency hearing loss

Independent variables	Male subjects (*n* = 278)		Female subjects (*n* = 420)	
	*p* value	OR (95% CI)	*p* value	OR (95% CI)
Age	<0.001*	1.170 (1.124–1.219)	<0.001*	1.157 (1.115–1.201)
Pack-years	0.12	1.015 (0.996–1.034)	0.61	1.009 (0.976–1.042)
Drinking habits	0.67	0.853 (0.412–1.769)	0.64	0.880 (0.515–1.505)
Noise exposure	0.01*	2.709 (1.322–5.548)	0.18	1.670 (0.786–3.548)
Hypertension	0.83	0.912 (0.394–2.108)	0.96	0.986 (0.538–1.807)
Diabetes	0.82	0.868 (0.252–2.987)	0.43	0.644 (0.217–1.912)
Dyslipidemia	1.00	0.999 (0.539–1.854)	0.03*	0.540 (0.315–0.925)
D4a	0.98	1.017 (0.330–3.136)	0.50	1.320 (0.587–2.968)

Statistical significance was set at *p* < 0.05 (*). *OR* Odds ratio, 95% CI: 95% confidence interval

**Table 3:** Influence of 12 major mtDNA haplogroups on hearing loss, after adjusting for age and known risk factors

mtDNA haplogroup	Male subjects (*n* = 278)		Female subjects (*n* = 420)	
	*p* value	OR (95% CI)	*p* value	OR (95% CI)
D4a	0.98	1.017 (0.330–3.136)	0.50	1.320 (0.587–2.968)
D4b	0.41	1.591 (0.525–4.823)	0.88	1.080 (0.404–2.885)
D5	0.86	1.184 (0.174–8.060)	0.29	0.461 (0.112–1.907)
G1	0.06	0.181 (0.031–1.071)	0.82	0.884 (0.307–2.549)
G2	0.48	1.950 (0.300–12.666)	0.77	0.769 (0.132–4.473)
M7a	0.79	0.852 (0.268–2.716)	0.56	1.323 (0.515–3.396)
M7b	0.11	0.357 (0.102–1.254)	0.05	2.460 (0.983–6.157)
A	0.01^*^	4.096 (1.327–12.643)	0.32	0.532 (0.154–1.842)
B4	0.60	0.776 (0.297–2.025)	0.42	1.385 (0.631–3.039)
B5	0.98	1.016 (0.215–4.794)	0.16	0.215 (0.025–1.851)
N9	0.84	1.158 (0.283–4.736)	0.02^*^	0.091 (0.012–0.712)
F	0.61	1.369 (0.415–4.520)	0.09	2.151 (0.880–5.257)
Others	0.71	0.867 (0.414–1.816)	0.73	0.904 (0.506–1.613)

All variables were examined using the multiple logistic regression analysis. Independent variables were age, pack-years, drinking habit, noise exposure, hypertension, diabetes, dyslipidemia, and each mtDNA haplogroup. Statistical significance was set at *p* < 0.05 (*). *OR* Odds ratio, 95% CI: 95% confidence interval.

## Discussion

Epidemiological studies showed that ARHL generally involves impaired hearing at high frequencies at the beginning of the disease [7, 8]. For the purpose of detecting early hearing loss with aging, subjects aged only 30‒64 years were enrolled and average hearing thresholds at higher frequencies were used for analysis in the present study. The evaluation of hearing at high frequencies may be useful as a screening method for ARHL in young individuals.

In recent years, it has been shown that normal aging is a multistep process that can be induced by ROS [15]. ARHL is an age-related disease, and oxidative damage in the cochlea reflects an age-related decline in antioxidant defenses and/or an increase in ROS levels and plays a crucial role in the development of ARHL [1]. ROS can affect both nuclear DNA (nDNA) and mtDNA. However, mtDNA is highly sensitive to oxidative damage, as it lacks “protective” histones and has a limited repertoire of available DNA repair pathways. As a result, mtDNA damage results in mitochondrial dysfunction, leading to an increase in ROS production, which elevates the accumulation rate of mtDNA mutations, further impairing respiratory chain function. Finally, the accumulation of somatic mtDNA mutations promotes apoptosis [15]. ROS also play a major role in cochlea degeneration. Sha et al. reported that outer hair cells at the basal turn of the cochlea are intrinsically more susceptible to free-radical damage than the cells at the apex turn of the cochlea [23]. These results are consistent with the results of epidemiological studies, which showed that at the beginning of the disease [7, 8]. Furthermore, there are differences in the degree of hearing impairment with aging among individuals. Therefore, individual differences in ROS production and antioxidant function are important when considering the development of ARHL. Individual differences in ROS production may be related to mutations in specific mtDNA and may be indicators of the risk of developing various systemic disorders [18, 20].

In the present study, we demonstrated that mtDNA haplogroup A is a risk factor for ARHL in male subjects and N9 as a protective factor in females. Haplogroup A is characterized by the mtSNP (single-nucleotide) m.8794 C > T polymorphism [22]. The polymorphism causes p.His90Tyr substitution, which is an amino acid substitution at a site that plays an important role in the proton translocation of the ATPase subunit 6 in ATP synthase in the electron transport chain [20]. Zhang et al. reported that the mtSNP m.8794 C > T mutation increases intracellular ROS levels [24], and Nishigaki et al. have reported that haplogroup A is a risk factor for atherothrombotic cerebral infarction [20]. mtDNA haplogroup N9 is mainly distributed in East Asia and is subclassified into N9a, N9b, and Y groups [22]. Each subgroup of haplogroup N9 has a specific mtSNP; for example, haplogroup N9a has m.12358 A > G, and haplogroup N9b has m.11016 G > A and m.13183 A > G. On the other hand, these subgroups N9 are associated with the following amino acid substitutions in common: m.11016 G > A (ND4:p.Ser86Asn), m.12358 A > G (ND5:p.Thr8Ala), and m.13183 A > G (ND5:p.Ile283Val) [18, 19]. Amino acid replacements in the ND4 and ND5 subunits might be related to lower ROS leakage [19]. It can be inferred that compared with other haplogroups, haplogroup N9 has a protective effect against age-related diseases because the reduced ROS leakage reduces mtDNA damage. In addition to m.12358 A > G, N9a has m.150 C > T, which is a specific mtSNP in the non-coding region of the mitochondrial genome. m.150 C > T has been reported to be associated with longevity [25]. Studies have reported that haplogroup N9a is associated with resistance to metabolic syndrome [25] and type 2 diabetes [18, 25], and haplogroup N9b is associated with resistance to myocardial infarction [19, 25]. According to the above, individuals belonging to haplogroup A, which is related to increased intracellular ROS levels, tend to develop ARHL. Contrarily, individuals of haplogroup N9, which is related to decreased ROS leakage, are less likely to develop ARHL. In the percentage of haplogroup M7b, there was a significant difference between the hearing loss and control groups in the present study. However, the multiple logistic regression analysis after adjusting for confounding factors influencing hearing showed that haplogroup M7b was not significantly associated with hearing. Therefore, the results of the present study do not indicate that haplogroup M7b is associated with ARHL development, consistent with the previous study. ^26^ Contrarily, Kato et al. suggested that haplogroup D4b may be one of the modifiers associated with the phenotypic expression of hereditary hearing loss in Japanese [26]. However, the present study did not show a significant correlation between the hearing loss at high frequency and haplogroup D4b after considering confounding factors. The difference may come from the differences in the subjects and the definition of hearing loss for detecting early hearing loss with aging.

ARHL is known to progress faster in males than in females [6]. The higher prevalence of hearing loss in males compared to females (Table 1) correlates well with previous studies [27, 28]. The prevalence of smoking history, drinking habit, noise exposure, and dyslipidemia were significantly higher in males. Accordingly, the prevalence of hearing loss was thought to be significantly higher in males, even though female subjects were significantly older than male subjects. The results of the present analyses in any haplogroup indicated that noise exposure significantly increased the risk of hearing loss only among male subjects. Several epidemiological studies have reported that noise exposure results in the progression of hearing loss only in males [5, 28–30]. Our findings are consistent with those of the previous studies, and hearing in male subjects might be more influenced by noise exposure or males may be more exposed to occupational noise [31]. Furthermore, dyslipidemia significantly decreased the risk of hearing loss among female subjects in our multiple logistic regression analyses (Table 2). Helzner et al. reported that higher TG levels are associated with poorer hearing sensitivity in males; however, hearing sensitivity was not associated with total cholesterol, LDL-C, and HDL-C in both males and females [32]. Gates et al. reported that there was no relationship between the cholesterol and TG levels and hearing in either gender [33]. The results of the present study indicated that dyslipidemia did not significantly lead to the progression of hearing loss. Because the subjects of the present study were relatively younger, dyslipidemia might not result in hearing loss, except its progression. The other variables did not influence hearing loss in the present study. This may be explained by that the effect of the disease on hearing loss may be less pronounced than that in the elderly. These considerations are uncertain and further detailed studies might be necessary to clarify the association between risk factors and hearing loss.

In this study, differences in the correlation between ARHL and mtDNA haplogroups depending on gender were observed. Some studies have reported that there were differences depending on gender, but the reason for gender differences was not clear. Haplogroup N9a is a protective factor for type 2 diabetes only in females [18], and haplogroup N9b is a protective factor for myocardial infarction only in males [19]. Furthermore, a study on the association between haplogroup A and atherothrombotic stroke suggested that haplogroup A is a risk factor only in females [20]. However, in these studies, gender differences in the influences of these haplogroups on various diseases were not revealed, which may be attributed to the difference in sex hormones and environmental factors, such as smoking [19].

In conclusion, multivariate analyses after considering confounding factors were performed to investigate the association between any mtDNA haplogroup and ARHL development. The results indicated that males in mtDNA haplogroup A were more likely to develop ARHL than males in other haplogroups, and females in haplogroup N9 were less likely to develop ARHL than females in other haplogroups. This suggested that the mtDNA haplogroup may be an indicator for future risk of morbidity associated with ARHL.
